# A comparative study of two percutaneous pinning techniques (lateral vs medial–lateral) for Gartland type III pediatric supracondylar fracture of the humerus

**DOI:** 10.1007/s10195-016-0410-2

**Published:** 2016-06-16

**Authors:** Kumar Prashant, Devendra Lakhotia, Tulsi Das Bhattacharyya, Anil Kumar Mahanta, Aakhil Ravoof

**Affiliations:** 1 Trauma Centre, Banaras Hindu University, Varanasi, UP 221005 India; 2 Institute of Medical Sciences and Research Center, Jagatpura, Jaipur, India; 3 Department of Orthopaedics, Gauhati Medical College and Hospital, Guwahati, Assam India; 4 Mysore Medical College and Research Institute, Mysore, India

**Keywords:** Supracondylar fracture, Humerus, Percutaneous fixation, Iatrogenic ulnar nerve injury, Randomized controlled study

## Abstract

**Background:**

The management of displaced supracondylar fracture of the humerus with closed reduction and percutaneous pin fixation is the most widely accepted method of treatment, but controversy continues regarding the pin fixation techniques. A prospective randomized controlled study was undertaken to compare the stability, functional outcome and iatrogenic ulnar nerve injury between lateral pin fixation and medial–lateral pin fixation.

**Material and method:**

Sixty-two patients with Gartland type III supracondylar fracture of the humerus were randomized into two groups—lateral pin fixation (*n* = 31) and medial–lateral pin fixation (*n* = 31). Primary assessment was performed for major loss of reduction and iatrogenic ulnar nerve injury. Secondary assessment included clinical outcome, elbow range of motion, radiographic measurements, Flynn grade, and complications.

**Results:**

There were two (6.5 %) iatrogenic ulnar nerve injury cases in the medial–lateral entry group and two (6.5 %) cases with mild loss of reduction in the lateral entry group. No major loss of reduction was observed in either of the groups. There was no statistically significant difference in change of Baumann angle, metaphyseal–diaphyseal angle, Flynn grade, carrying angle, and the total elbow range of motion (*P* < 0.05) between the two groups.

**Conclusions:**

Lateral pin fixation offers similar functional and radiological outcome and almost equal mechanical stability compared with medial–lateral pinning without the risk of iatrogenic ulnar nerve injury.

**Level of evidence [OCEBM 2011]:**

Level 2.

## Introduction

Supracondylar humerus fractures constitute 60–65 % of all the fractures around the elbow joint, with a peak incidence between 4 and 7 years of age in children [1]. The main complications associated with supracondylar fractures are malunion, ischemic contracture and neurovascular damage [2, 3]. Of the methods described for the treatment of displaced extension-type supracondylar humeral fractures, closed reduction with percutaneous pin stabilization is the current preferred method of treatment [1]. However, controversy persists between lateral or crossed medial and lateral pin fixation techniques [4].

Two major complications associated with percutaneous pinning are iatrogenic ulnar nerve palsy and loss of reduction, resulting in cubitus varus deformity [5, 6]. The optimal pin configuration that provides adequate stability of the fracture and minimizes the risk of iatrogenic neurovascular injury is still a matter of discussion.

The advantage of medial−lateral entry pin fixation is probably increased biomechanical stability, although iatrogenic ulnar nerve injury may result from placement of the medial pin [2, 4, 7]. Conversely, the advantage of lateral entry pin fixation is avoidance of iatrogenic ulnar nerve injury, although the construct may be less stable biomechanically [2, 8–10]. A few studies reported that there is no significant advantage of cross pins in comparison to lateral pins [11, 12].

The aim of this study was to compare the efficacy in terms of stability, functional outcome and iatrogenic ulnar nerve injury between lateral entry pin fixation and medial–lateral entry pin fixation of completely displaced (type-III) extension supracondylar fractures of the humerus in children. The null hypothesis was that there would be no difference between the pin fixation techniques in terms of major loss of reduction or iatrogenic ulnar nerve injury.

## Materials and methods

We conducted a prospective, single-blinded randomized control trial in the Department of Orthopaedics, Gauhati Medical College and Hospital, Guwahati, Assam, India for a period of one year, after obtaining ethical committee approval. Full written informed consent was taken from parents/legal guardian before participating in this study. Inclusion criteria for this study were aged between 3 and 12 years, closed Gartland type III supracondylar humeral fracture [13], duration of injury <4 days, and competent neurological and vascular status of the affected limb. Exclusion criteria were duration of injury >4 days, inability to take part in postoperative rehabilitation, open fractures, medical contraindications to surgery, fracture requiring open reduction or neurovascular exploration, previous ipsilateral elbow fracture, and floating elbow injury.

A total of 216 patients with supracondylar humerus fractures were admitted to the orthopedic wards either through the outpatient department or emergency services. Of the 216 patients, 140 were excluded from the present study as they did not fulfill the inclusion criteria. These included compound fractures (10 cases), aged >12 years (12 cases), were not fit for surgery/refused surgery (15 cases), were associated with ipsilateral forearm fractures (6 cases), or were being treated conservatively for Gartland I and II fractures (46 cases). The remaining 76 patients were enrolled in the study. The method of patient selection for lateral entry or medial−lateral entry was random, using a computer-generated randomization table from http://www.randomization.com. The seed for the random number generator was obtained from the clock of the local computer and was printed at the bottom of the randomization plan. Fourteen patients were excluded from the final analysis because of lost to follow-up. Our analysis included 62 patients who were followed up for at least 6 months at 1, 3, 6, 14, 18, and 24 weeks and then at 3-month intervals.

All the children with suspected supracondylar fractures of the elbow were assessed for vascular and neurological status. Anteroposterior and lateral radiographs were performed. All displaced supracondylar fractures were admitted and the injured elbow was immobilized in an above-elbow splint with the elbow at 30°–45° of flexion and limb elevation. Pulseless viable limbs [absent radial pulse because of complete transaction, intimal tear or compression (temporary compression or reversible spasm) of brachial artery, but hand viable because of good collaterals around elbow] were also included in the study. In all such cases a vascular surgeon was present for the surgery but radial pulsation appeared in all cases after close reduction and pinning. Therefore, brachial artery exploration was not needed for any of our cases.

**Fig. 1:**
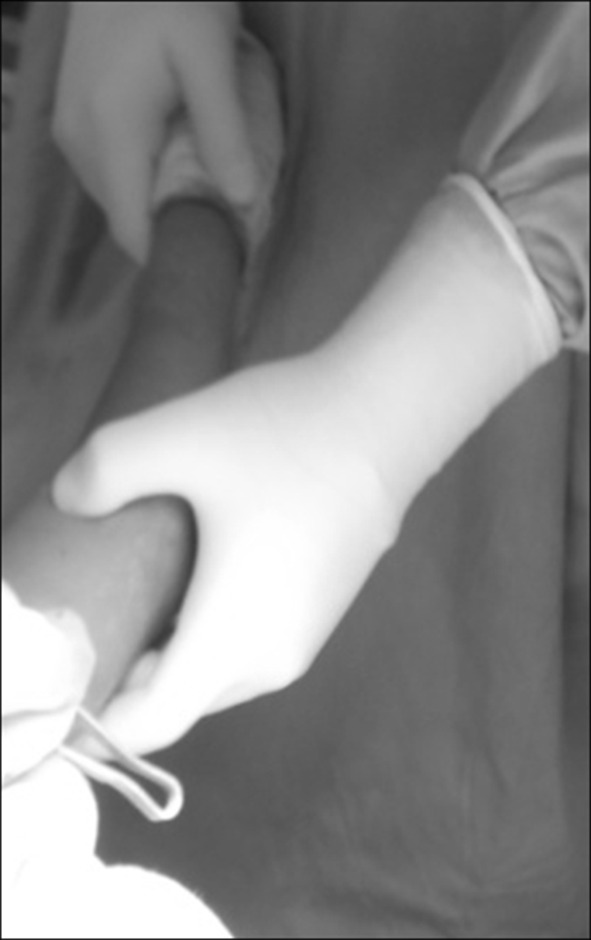
Close reduction technique

**Fig. 2:**
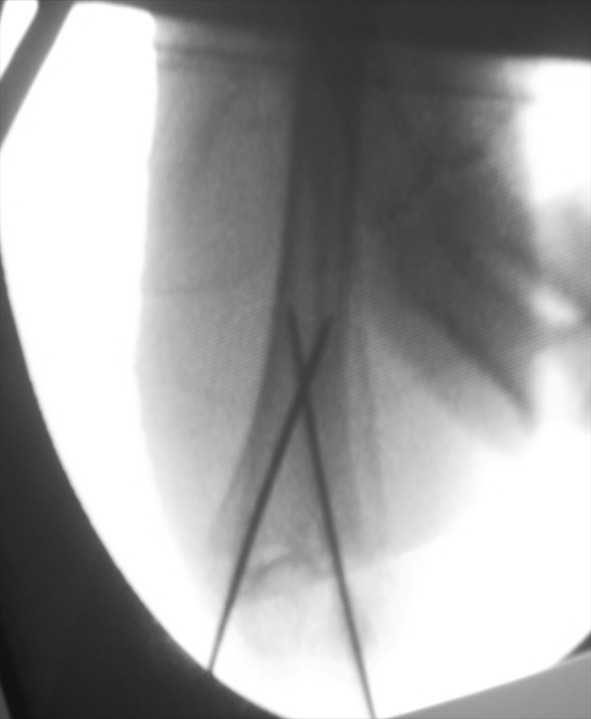
Reduction confirmation in A/P view

**Fig. 3:**
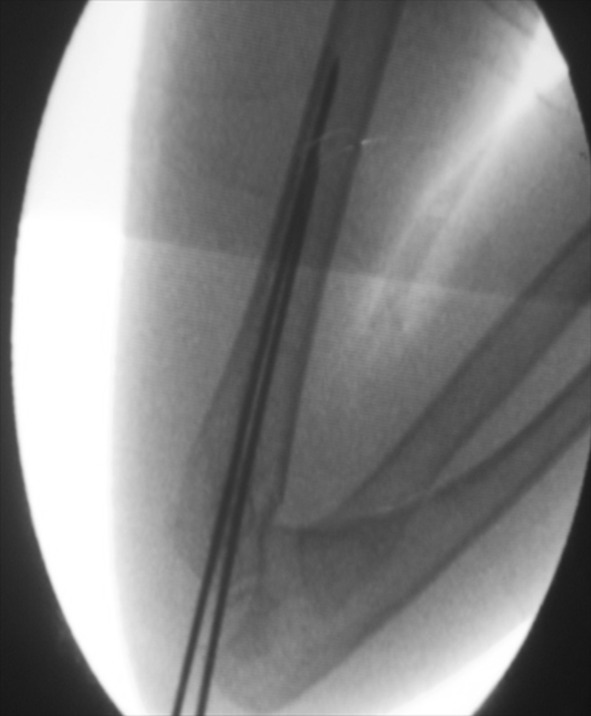
Reduction confimation in Lat. view

Surgical techniques were standardized in terms of pin location, pin size (weight <20 kg size 1.5 mm; >20 kg size 2 mm), stability on the table and the position of the elbow for pin placement. Surgery was performed by a senior orthopedic surgeon who was well trained in this technique. General anesthesia was used for all patients with the injured upper limb on the side of the table. The injured elbow was placed on the plate of the image intensifier which was adequate for the surgery due to the small size of the elbow. Closed reduction was performed and confirmed by the image intensifier. First, longitudinal traction was applied with the elbow in hyperextension and the forearm in supination (Fig. 1). While the traction was maintained, the medial or lateral displacement was corrected by applying a valgus or varus force at the fracture site. The posterior displacement of the distal fragment was then corrected by applying a force to the posterior aspect while the elbow was gently hyperflexed and the elbow was secured in hyperflexion, and the reduction was confirmed by the image intensifier. The medial pin was placed directly through the apex of the medial epicondyle. The lateral pin was placed at the center of the lateral epicondyle. For the lateral fixation technique, two or three pins were inserted from the lateral aspect of elbow across the lateral cortex to engage the medial cortex keeping the elbow in hyperflexion. Pins were placed either in parallel or divergent configuration with adequate separation at the fracture site. For the medial−lateral fixation technique, first the lateral pin was inserted from lateral cortex to engage the medial cortex keeping the elbow in hyperflexion. The elbow was then extended to <90° and the ulnar nerve rolled back with the opposite thumb and the medial pin was inserted to engage the lateral cortex with the elbow in <90° of flexion. The pin configuration was considered to be acceptable if one pin was placed in the lateral column and another in the central or medial column. If this was not achieved, we realigned the configuration by changing the pin placement. In the coronal plane, the pins were placed at an angle of 30° with the long axis of the humerus. After the pins were placed, the elbow was extended and the carrying angle was measured and compared with that on the non-affected side. The adequacy and stability of the reduction were checked under image intensification (Figs. 2, 3). The pins were bent to prevent migration and cut off outside the skin to allow removal in the outpatient clinic.

A single preoperative parenteral dose of cefuroxime was given at the time of induction and postoperatively, and oral cefuroxmime was given for three days at the time of discharge. Postoperatively, the extremity was placed in a well-padded posterior splint with the elbow flexed to 90°. Any patients with immediate postoperative ulnar nerve deficit were investigated and the pin was placed in another location. For all patients, immediate postoperative radiographs were taken to determine the maintenance of the reduction. The operated limb was elevated and carefully observed at regular intervals for any neurovascular deficit.

During follow-up in the outpatient department, clinical-radiological evaluation was performed for maintenance of reduction (at first follow-up) and functional outcome, which included passive range of motion, measurement of carrying angle, Baumann angle, metaphyseal–diaphyseal (MD) angle, neurovascular status, superficial and deep infection, and the necessity to re-operate. Clinical evaluation was graded according to carrying angle and elbow range of motion using the criteria of Flynn et al. [14]. Radiographic evaluation was performed by anteroposterior and true lateral view at 1, 3, and 6 weeks and at 3 and 6 months. In the third week, the pins were removed without anesthesia. At 3- and 6-month follow-up, the children were evaluated for full function, minor limitation of function and major loss of function.

The final results were graded as excellent, good, fair and poor, according to the loss of range of motion and loss of carrying angle using the criteria of Flynn et al. Loss of reduction was graded by the loss of Baumann angle using the classification of Gordon et al. [5]. Statistical screening of treatment effects was measured by relative risk reduction, absolute risk reduction with adjustment for a small sample size and confounders in the study. The Fisher exact test and unpaired *t* test were applied to check for the presence of a significant difference in outcome variable between the two groups. The software InStat version 3.10, 32 bits from GraphPad was used in the statistical analysis. A *P* value of <0.005 % was considered significant.

## Result

The mean age of the patients was 8.4 years. The mean age in the lateral pin group was 8.25 years and 8.55 years in the medial–lateral pin fixation group. In the lateral entry group, 23 were male and 8 were female, whereas in the medial–lateral entry group, 22 were male, and 9 were female. The commonest cause of injury was falling while playing (64.51 %), followed by fall from a tree (27.41 %) and fall from a bicycle (8.06 %). Involvement of the left side was 77.4 % and 22.6 % for the right side. Left and right side involvement was 83.87 and 16.13 % in the lateral entry group and 70.9 and 29.1 % in the medial−lateral entry group, respectively. At the time of presentation, the radial pulse was weak in 54.83 %, normal in 37.09 %, and absent with the viable hand in 8.06 %. In this study, the frequency of posteromedial and posterolateral injuries was 80.65 % and 19.35 %, respectively. The frequency in the lateral entry group was 87.1 and 12.9 %, respectively and 74.9 and 25.81 % in the medial–lateral entry group, respectively. The average delay in reporting the injury was 1.79 ± 0.54 days. The average delay between the day of injury and day of the operation was 2.3 days. In the lateral entry group, the average delay was 2.25 days and 2.35 days in the medial−lateral entry group. The average hospital stay was 2.41 days with a minimum and maximum duration of two and four days, respectively. In the lateral entry group, the average hospital stay was 2.32 days and 2.51 days in the medial−lateral entry group. Mean duration of follow-up was 35.29 weeks with a minimum duration of 24 weeks and maximum duration of 64 weeks. In the lateral entry group, the mean duration of follow-up was 32.64 weeks and 34.12 weeks in the medial−lateral group. There were no significant differences (*P* > 0.05) between groups with regard to any of these variables (Table 1).

**Table 1:** Analysis of carrying angle loss, Baumann angle loss, MD angle loss and range of motion loss at 6-month follow-up

Parameters	Lateral entry group (mean ± SD)	Medial–lateral entry group (mean ± SD)	*P* value
Loss of carrying angle^a^	4.12 ± 2.10	3.80 ± 2.02	0.54
Loss of Baumann angle^a^	4.74 ± 1.29	4.99 ± 0.87	0.50
Loss of MD angle^a^	2.34 ± 0.65	2.21 ± 0.61	0.39
Loss of range of motion^a^	8.03 ± 3.65	7.54 ± 1.89	0.51

^a^Values are given as the mean and SD

**Fig. 4:**
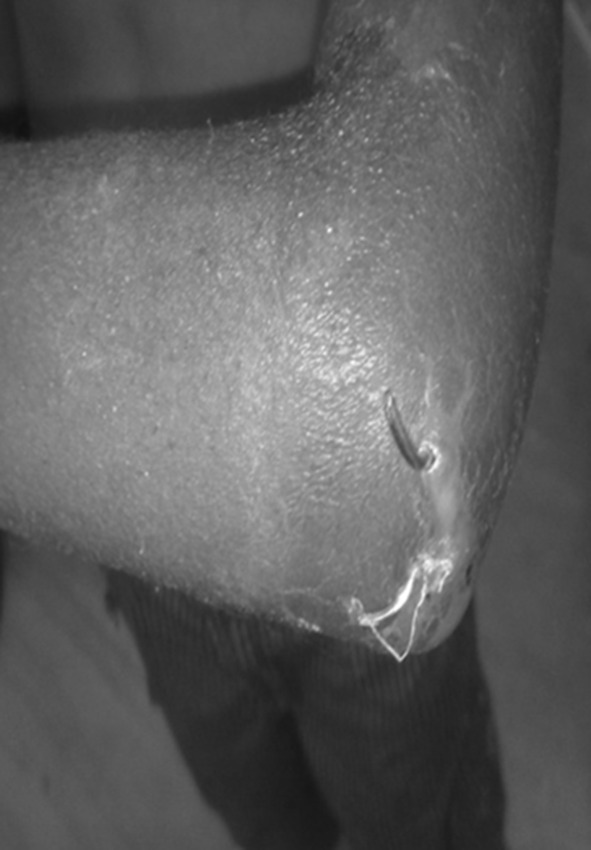
Pin tract infection

Postoperative complications like pin tract infection were found in four cases (three in the lateral entry group and one in the medial−lateral entry group) but all infections were superficial only (Fig. 4). There were two cases of iatrogenic ulnar nerve palsy following medial pinning (6.5 %) in the medial–lateral entry group—one case had paraesthesia along the ulnar nerve distribution, which subsided spontaneously within three weeks, and the other case had both motor and sensory deficits but complete neurological recovery occurred after four months.

No patient in either group had a major loss of reduction. There was a mild loss of reduction in two cases and both were in the lateral entry group. Although radiological and clinical union occurred within a similar time period without any residual deformity, the loss of both the range of motion and the carrying angle was greater in these two patients compared to those without loss of reduction. However, there were no significant differences (*P* > 0.05) between groups regarding change in the Baumann angle, MD angle, carrying angle, or total elbow motion (Table 1). According to Flynn criteria, the final result was excellent in 79.03 % and good in 20.97 % of cases. The result for the medial−lateral entry group was excellent in 83.87 % and good in 16.12 % cases, and the result for the lateral entry group was excellent in 74.19 % and good in 25.82 % (Table 2).

**Table 2:** Comparison of variables

Variables	Lateral entry (*n* = 31)	Medial−lateral entry (*n* = 31)	*P* value
Sex distribution^a^			
Male	23	22	1.000
Female	8	9	
Mean age of the patient (years)^b^	8.25 ± 2.26	8.55 ± 2.33	0.314
Side affected^a^			
Left	26 (83 %)	22 (71 %)	0.362
Right	5 (17 %)	9 (29 %)	
Hospital stay (days)^b^	2.32 ± 0.50	2.51 ± 0.64	0.381
Fracture type^a^			
PM	25 (87 %)	23 (74 %)	0.762
PL	6 (13 %)	8 (26 %)	
Average delay from injury to surgery (days)^b^	2.25 ± 0.68	2.35 ± 0.66	0.447
Average follow-up (weeks)^b^	35.29 ± 9.84	33.529 ± 10.36	1.000
Pin tract infection^a^	3 (9.6 %)	1 (3.2 %)	0.612
Iatrogenic ulnar nerve injury^a^	0 (0 %)	2 (6.5 %)	0.491
Functional results (Flynn grading)^a^			
Excellent	23 (74.19 %)	26 (83.87 %)	0.533
Good	8 (25.82 %)	5 (16.12 %)	

*PM* posteromedial, *PL* posterolateral, *MD* metaphysio-diaphyseal

^a^Values are given as the number of patients

^b^Values are given as the mean and SD

## Discussion

The ideal treatment for completely displaced (type-III) extension supracondylar fractures of the humerus in children is closed reduction and percutaneous pin fixation. However, controversy regarding the optimal technique, whether lateral or crossed medial–lateral pin fixation is still debatable.

According to earlier studies, the advantage of medial–lateral entry pin fixation is increased biomechanical stability [7, 15], although iatrogenic ulnar nerve injury may result from placement of the medial pin [4]. Conversely, the advantage of lateral entry pin fixation is avoidance of iatrogenic ulnar nerve injury, although the construct may be less stable biomechanically [10, 11, 16, 17] and failure to provide torsional stability, for which some have suggested adding a third medial pin [11, 18]. A biomechanical study by Zionts et al. [7] demonstrated that crossed pinning is more stable than lateral pinning in rotational testing as well as varus and valgus loading. However, a study by Skaggs et al. [10] demonstrated no clinical difference in stability between crossed and lateral pins.

The average loss of the carrying angle, Baumann angle, M–D angle and range of motion in the lateral pinning cases in our study may be related to a comparatively less stable construct with two lateral pins compared to crossed medial–lateral pins. According to the classification by Gordon et al. [5], the mild loss of reduction in two cases of lateral entry group in our study suggest that lateral entry is biomechanically weaker. Although radiological and clinical union occurred in a similar time period without any residual deformity, the loss of both the range of motion and the carrying angle was greater in these two patients, compared to those without loss of reduction. In a recent analysis of the two techniques by Lee et al. [19], the loss of reduction in the lateral entry group was 0–11.8 %. An older study by Kallio et al. found a reduction of 14 % [12], a study by Davis et al. found 29 % [20], while a study by Skaggs et al. found 0 % [10]. The risk of loss of reduction after lateral entry pin fixation can be minimized by following proper pin placement technique, with divergent pins, pins that engage the lateral and central columns, and the use of a third lateral pin if needed.

The reported risk of iatrogenic ulnar nerve injury from medial entry pin fixation has been found to range from 1.4−15.6 % [6, 21], and depends on the technique of pin insertion. In a recent trial by Lee et al. [19], the risk of iatrogenic ulnar nerve injury was 0–6.8 %. In our study, the risk was 6.5 % (2 cases) in the medial–lateral entry group—one case showed only paraesthesia along the ulnar nerve distribution, which subsided spontaneously within one week while the other case of nerve palsy with both motor and sensory deficits, showed complete neurological recovery after 4 months. The incidence of ulnar nerve injury in our study was low because of precautions such as inserting the lateral pin first, avoiding hyperflexion of the elbow during medial pin placement and by retracting the nerve more posteriorly by the digital method before medial pin insertion. The risk of iatrogenic ulnar nerve injury can be further reduced with a mini medial incision as reported by Kocher et al. [2] and with extension of the elbow during medial pin placement. Iatrogenic ulnar nerve injuries associated with medial pin fixation resolve after replacement of the medial pin at a new location [21], as occurred in our two cases.

In our study, the difference with regard to the loss of range of movement between the two groups was statistically insignificant (*P* = 0.51), with both groups showing an excellent or good range of movements. The functional outcome following medial and lateral pinning was excellent in 83 % and good in 17 % of cases. There were no poor results, while cases treated with lateral pinning showed 74 % excellent and 26 % good results with no poor results. Similar results were shown by Kocher et al. [2], Mostafavi and Spero [22], and Aronson and Prager [23]. The difference in functional outcome between the two groups in our study was not statistically significant (*P* = 0.53).

One of the strengths of this study was being a prospective randomized clinical trial with the patients randomized at the time of fracture treatment. Furthermore, both the lateral entry and the medial–lateral entry techniques were standardized in terms of pin size, pin location, and the position of the elbow for medial pin placement. Full clinical and radiographic evaluation was performed at regular intervals. The major limitation of the study was the small number of cases in each group. A randomized controlled trial (possibly triple blind) involving a large number of patients with long-term follow-up is clearly needed to clarify the differences between the two techniques.
